# Relationships among sense of coherence, resources, and mental health in urban and rural residents in Japan

**DOI:** 10.1186/1471-2458-12-1107

**Published:** 2012-12-23

**Authors:** Yoko Sumikawa Tsuno, Yoshihiko Yamazaki

**Affiliations:** 1 Faculty of Nursing, Toho University, Tokyo, Japan; 2 Faculty of Social Welfare, Nihon Fukushi University, Aichi, Japan

**Keywords:** Sense of Coherence, Salutogenic model, Regional differences, Coping resources, Mental health, Population-based study, Japan

## Abstract

**Background:**

The salutogenic model states that coping resources are defined within sociocultural and historical contexts and that various social and historical factors influence the availability of such resources. Though previous studies have suggested the need for an interregional comparison of psychological and social resources, few studies have undertaken such an investigation. The aim of this study is to investigate the associations among coping resources, sense of coherence (SOC), and health status in a comparison of urban and rural residents.

**Methods:**

General residents (aged 30–69 years) in two areas were targeted for the current study. Through a random sampling selection, 1,000 residents from each area were picked, and an anonymous questionnaire was mailed to each resident. Ultimately, 269 and 363 valid responses from the urban and rural areas, respectively, were analyzed. SOC, both social and psychological resources, and mental health were assessed. To examine relationships between SOC and resources associated with mental health, mental health was defined as a dependent variable. Hierarchical multiple regression was conducted with variables entered from sociodemographic characteristics, social and psychological resources, and SOC.

**Results:**

Regarding regional characteristics, social capital and participation in community activities were significantly greater in the rural area than in the urban area. Urban residents reported significantly higher self-esteem and optimism than rural residents. SOC showed the most significant association with mental health in both areas. Mental health was significantly associated with physical activity limitations and life stressors in both areas. However, the associations were weakened when social and psychological resources and SOC were added, which demonstrated their buffering effect on the negative influence of life stressors on health. When SOC was added, the association of self-esteem with mental health disappeared in both areas, but optimism in the urban area and humor in the rural area were directly and independently associated with mental health.

**Conclusions:**

Our findings indicate that resources available to urban and rural residents are characteristic of the area where they live, and that social and psychological resources as well as SOC are associated with better mental health. Possible directions for health support strategies, reflecting regional characteristics, are suggested.

## Background

Traditional medicine is based on a pathogenic perspective that questions how diseases are created, and therefore, focuses on risk factors for diseases. Knowledge has been accumulated on ways to alleviate or eliminate these factors. In contrast, the salutogenic model theorized by Antonovsky looks at how health is restored, maintained, and promoted. It attempts to elucidate salutary factors, and to support and strengthen them [1]. However, it is important that pathogenic and salutogenic models develop complementarily [1]. The salutogenic model is highly valued as a basic theory for health promotion [2]. It consists of a coping ability termed “sense of coherence (SOC)” and coping resources or “generalized resistance resources,” and is based on the three following assumptions. First, in the face of stressors and the resultant strain, SOC tries to cope by mobilizing generalized resistance resources. Second, the success or failure of this coping affects health, with successful coping producing positive effects on health. Third, the success or failure of coping depends on the richness of coping resources and the strength of SOC.

A person with strong SOC is better able to cope with stressors, which has a positive effect on health. Thus, SOC is also considered stress coping ability. Because SOC can strongly predict health outcomes, a number of studies have been conducted on its buffering effect on stress [3-8] and its predictive ability related to health status [9-15] and well-being [3,16].

In the salutogenic model, the richness of coping resources as well as the strength of SOC are considered important to the success or failure of stress coping. In general, coping resources used in the stress coping process are divided into psychological and social. Psychological resources include personality tendencies such as optimism and sense of humor as well as self-concept, which includes factors such as self-esteem and self-efficacy. Social resources include social support and social networks. A number of previous studies have examined the association of self-esteem with SOC [11,16,17]. This author and her colleagues found that sense of humor orientation was associated with SOC only in a rural area compared with an urban area [18]. Previous studies have reported that social support and participation in regional activities are associated with SOC [18,19]. However, few studies have examined the association between social capital and SOC.

A number of studies have also been conducted on the relationship of coping resources with well-being and health outcomes, indicating the importance of such resources [20]. Other studies have pointed out that a lack of coping resources can be a risk factor in the stress coping process [21]. Thus, there is a growing interest in exploring the effects of coping resources.

Previous studies have often treated SOC as one type of psychological coping resource, such as self-efficacy or self-esteem [14,16,17]. However, SOC in the salutogenic model is defined as the ability to mobilize coping resources in stress coping, and therefore should be distinguished from coping resources. Because SOC is the ability to mediate between coping resources and health, it is also considered a ‘health promoting resource’ [22] and has drawn attention from health promotion and empowerment research. Furthermore, SOC has been found relevant in resource theories [20], with regard to the ability to use coping resources as well as the usefulness of such resources.

The salutogenic model posits that coping resources are defined within sociocultural and historical contexts and that various social and historical factors influence the availability of these resources. For example, an international comparison of personality characteristics, which are considered to be psychological resources, points to the individualistic tendency of Western people and the collectivistic tendency of Japanese individuals [1]. Coping resources are different depending on the country as well as regions within the country, such as urban and rural areas. Various sociocultural differences exist in these areas, including geographical conditions and industrial structures [23].

Previous studies have suggested that an interregional comparative investigation is needed. However, few studies have actually investigated both psychological and social resources in different regions. A study by this author and her colleagues demonstrated that SOC-related factors were different between urban and rural areas. For example, economic status was correlated with SOC in urban but not rural residents, and ties with relatives and humor were correlated with SOC only in rural residents [18]. Therefore, for the purpose of providing support for residents’ health, it is important to compare regions with different sociocultural backgrounds. However, it is not only important to make such a comparison, but also to identify the meaning for the generation of SOC.

The purposes of this study are to 1) distinguish social and psychological coping resources, 2) examine the availability of both types of resources, and 3) examine associations among these coping resources, SOC, and health status. By comparing urban and rural residents, the current research also attempts to examine whether regional differences influence social and psychological resources associated with health, and their relationships with SOC.

## Methods

### Research respondents and survey method

The general residents of ‘A ward’ (urban A) of Tokyo and general residents of ‘B city’ (rural B) of Akita prefecture were chosen for the urban respondents and rural respondents, respectively. Urban A was a metropolitan area with a population of 297,989 and 170,714 households with an average of 1.75 persons per household in 2006. Rural B was a rural, mountain village area with a population of 40,382 and 14,815 households, with an average of 2.73 persons per household in 2006.

Men and women aged from 30 to 69 were targeted for the current study. Through a two-step random sampling selection, 1,000 residents from each area (totaling 2,000) were picked, and an anonymous questionnaire was mailed to each resident. The survey was administered in September 2006. As a result, 269 valid responses from urban A (valid response rate: 26.9%) and 363 valid responses from rural B (valid response rate: 36.3%) were used for the final analysis.

This study was approved by the Graduate School of Medicine of the University of Tokyo Research Ethics Committee.

### Variables and measures

The Japanese version of Antonovsky’s SOC scale [2], which has 13 questions scored on a 5-point Likert scale, was used (Cronbach’s α = 0.85) [24]. The SOC scale has been translated into almost 40 languages, and 1,300 research papers using the scale have been published. Its reliability and validity have been examined and verified [10,25,26].

Data on sociodemographic characteristics were collected, including age, gender, physical activity limitations, education, economic status, marital status, and employment status.

Three social resource variables were incorporated: social capital, social support, and participation in community activities. The current study considered social capital to be an important resource that reflects regional characteristics. Therefore, it was included as one of the items to be surveyed. Social capital is the degree of connectedness and social relations in a residential area. The concept of social capital is composed of networks, trust, and norms. To measure the individual level of social capital, which is a subjective view of one’s environment, a 6-item scale was used scored on a 5-point Likert scale (Cronbach’s α = 0.76) [27]. The social support scale comprised 6 items. Five items were created based on Noguchi’s [28] scale of emotional and instrumental support, and another item regarding the existence of someone in whom one has confidence was added (Cronbach’s α = 0.79). Questions on participation in community activities included participation in “local organizations,” “club activities,” “helping others with celebrations or funerals,” “volunteer activities,” and “political activities.” Answering yes to any of these items was defined as participation in regional activities, and participating in none of these items was defined as no participation in community activities.

Three psychological resource variables were incorporated: self-esteem, humor, and optimism. The Japanese version of Rosenberg’s (1965) Self-Esteem Scale [29], which has 10 questions scored on a 5-point Likert scale, was used (Cronbach’s α = 0.85). Because there are considerable cultural differences in sense of humor and its usage, humor was measured by a sense of humor scale developed in Japan [30] that questions views and attitudes toward humorous matters. It consists of three factors: “supportive humor,” “playful humor,” and “aggressive humor.” The current study adopted the 8 items scored on a 5-point Likert scale of “supportive humor” (Cronbach’s α = 0.89). Supportive humor represents the intention to encourage and support oneself and others, and has been found to be associated with mental health. The Life Orientation Test by Scheier et al. [31] is a representative scale that measures optimism. The Japanese revised version [32] was used, which comprises 6 items scored on a 5-point Likert scale (Cronbach’s α = 0.62).

Life stressors were assessed by 7 items on “work,” “income and family budget,” “housing,” “family,” “reasons for living and future hope,” “physical strength and health,” and “life in old age.” Questions were asked regarding the degree to which respondents felt worried or anxious about these items.

Mental health was measured by Goldberg’s 12-item General Health Questionnaire (GHQ) [33]. The GHQ is a measure of current mental health. It has been widely applied to the general population as a scale for measuring mental health [34], although it is a screening test for psychiatric symptoms. The total score for the 12 items, rated on a 4-point Likert scale (0–3) was calculated (Cronbach’s α = 0.88).

### Statistical analysis

Urban A and rural B were separately analyzed for the regional comparison. All scores were added up, and regional differences were determined. The chi-square test was used for cross tabulation, with t-tests and the Mann–Whitney U-test used to examine differences between two groups of continuous data (0.05 level of statistical significance).

To examine associations between SOC and different categories of resources, multiple regression analysis was conducted with SOC as a dependent variable and variables entered from each category of demographic characteristics, social resources, and psychological resources as independent variables. Variables significantly associated with SOC and the explanatory strength of demographic characteristics, social resources, and psychological resources were examined.

To examine associations between SOC and resources related to mental health, mental health was defined as a dependent variable. Demographic characteristics and life stressors were defined as independent variables in Model 1. Social resources and psychological resources were added as independent variables in Model 2. Furthermore, hierarchical multiple regression was conducted with SOC as an independent variable in Model 3.

The statistical package SPSS 19.0 (IBM Japan Ltd., Tokyo, Japan) was used for data analysis.

## Results

### Characteristics of research respondents (Table 1)

**Table 1:** Characteristics of research respondents

	**Urban A**				**Rural B**				**p Value for comparison**
	**n**	**Mean/n**		**SD/%**	**n**	**Mean/n**		**SD/%**	
Age(year)	268	49.8	±	11.4	361	52.3	±	10.0	.004
Gender	267				361				
Female		152		(56.9%)		185		(51.2%)	.158
Male		115		(43.1%)		176		(48.8%)	
Physical activity limitations	266				359				
No limitation at all		111		(41.7%)		129		(35.9%)	.400
Very little limitation		106		(39.8%)		172		(47.9%)	
Some limitation		40		(15.0%)		43		(12.0%)	
Severe limitation		9		(3.4%)		15		(4.2%)	
Education	260				362				
Junior high school		14		(5.4%)		61		(16.9%)	<.001
High school		65		(25.0%)		196		(54.1%)	
Junior or technical college		73		(28.1%)		72		(19.9%)	
Four-year university or graduate school		108		(41.5%)		33		(9.1%)	
Economic status	263				361				
Barely sufficient for subsistence		12		(4.6%)		49		(13.6%)	<.001
No difficulties with subsistence		59		(22.4%)		117		(32.4%)	
Can afford some common amenities		141		(53.6%)		168		(46.5%)	
Economically comfortable		51		(19.4%)		27		(7.5%)	
Marital status	262				362				
Married		178		(67.9%		291		(80.4%)	<.001
Unmarried		84		(32.1%)		71		(19.6%)	
Employment status	262				359				
Employed		240		(91.6%)		322		(89.7%)	.423
Unemployed		22		(8.4%)		37		(10.3%)	
SOC score (range 13–65)	269	42.6	±	8.1	363	41.4	±	7.8	.058
Social capital (range=6-30)	263	19.8	±	4.4	358	22.9	±	4.1	<.001
Social support (range=0-6)	268	4.2	±	1.9	361	4.0	±	1.9	.125
Community activities	268				356				
Participation		107		(39.9%)		279		(78.4%)	<.001
None		161		(60.1%)		77		(21.6%)	
Self-esteem (range=10-50)	268	35.0	±	6.8	360	31.7	±	6.0	<.001
Humor (range=8-40)	269	26.6	±	6.1	360	26.0	±	6.6	.255
Optimism (range=6-30)	268	19.0	±	3.5	361	17.4	±	3.3	<.001
Life stressors (range 0–14)	268	7.5	±	3.4	360	8.3	±	3.4	.004
Mental health (GHQ) (range 0–36)	269	13.3	±	5.5	363	13.6	±	5.5	.506

The average age of respondents from urban A was 49.8 (SD = 11.4) years, and that of rural B was 52.2 (SD = 10.0) years. There was no regional difference with regard to physical activity limitations. Education and economic levels were significantly higher (p < .001) in urban A. The rate of married respondents was significantly higher (p < .001) in rural B (80.4%) compared with urban A (67.9%).

As for social resources, there was no significant difference between the two areas with regard to social support. However, social capital and participation in community activities were significantly greater (p < .001) in rural B. As for psychological resources, urban A residents reported significantly higher (p < .001) self-esteem and optimism than rural B residents. There was no significant difference between the two areas with regard to humor.

No significant difference was found between the two areas related to SOC and mental health.

### Associations of SOC and resources (Table 2)

**Table 2:** Associations of SOC and resources

	**Urban A (n=363)**		**Rural B (n=269)**	
	**β**	**p Value**	**β**	**p Value**
Sociodemographic characteristics				
Gender (1=Male, 0=Female)	-.09	.125	.10	.052
Age (years)	.35	<.001	.19	<.001
Physical activity limitations (1 No limitation at all~4 Severe limitation)	-.20	<.001	-.25	<.001
Education				
Junior high school (ref)				
High school	.07	.593	.06	.407
Junior or technical college	.10	.434	.12	.080
Four-year university or graduate school	.11	.398	.03	.636
Economic status (1 Barely sufficient for subsistence~4 Economically comfortable)	.26	<.001	.22	<.001
Marital status (1=Married, 0=Unmarried)	.12	.027	-.03	.614
Employment status(1=Employed, 0=Unemployed)	.08	.183	.03	.505
Adjusted R^2^	.29		.15	
Social resources				
Social capital	.14	.019	.29	<.001
Social support	.15	.012	.12	.025
Community activities (1=Participation, 0=None)	.14	.028	.03	.546
Adjusted R^2^	.06		.11	
Psychological resources				
Self-esteem	.60	<.001	.48	<.001
Humor	.04	.458	.06	.198
Optimism	.13	.018	.07	.219
Adjusted R^2^	.46		.29	

According to multiple regression analysis, SOC score as dependent variable.

Age, physical activity limitations, and economic status showed significant associations with SOC in both areas. In urban A, being married (β = .12, p = .027) was also significantly associated with SOC.

As for social resources, social capital and social support were significantly associated with SOC in both areas. Social capital was more strongly associated in rural B (β = .29, p < .001) than in urban A (β = .14, p = .019). Participation in community activities was significantly associated with SOC in urban A (β = .14, p = .028), but not in rural B.

As for psychological resources, self-esteem (urban A: β = .60, p < .001; rural B: β = .48, p < .001) was most significantly associated with SOC in both areas. Optimism was significantly associated with SOC in urban A (β = .13, p = .018), but not in rural B. No significant association of humor with SOC was found in the urban or rural area.

Adjusted R^2^ of sociodemographic characteristics was stronger in urban A (adjusted R^2^ =.29) than in rural B (adjusted R^2^ = .15), but that of social resources in rural B (adjusted R^2^ =.11) was stronger than in urban A (adjusted R^2^ = .06). Psychological resources had the strongest explanatory power (urban A: adjusted R^2^ = .46; rural B: adjusted R^2^ = .29) on SOC in both areas.

### Resources associated with mental health

In urban A, results of hierarchical multiple regression showed that mental health was significantly associated with age, physical activity limitations, economic status, and life stressors in Model 1. With social and psychological resources added to Model 2, its significant association with economic status disappeared, but mental health was significantly associated with self-esteem and optimism. With SOC added to Model 3, the significant association of mental health with optimism was unchanged, but the significant association with self-esteem disappeared. There was a significant association between SOC and mental health.

In rural B, mental health was found to be significantly associated with physical activity limitations and life stressors in Model 1. In Model 2, the significant associations with physical activity limitations and life stressors were unchanged, and mental health also showed significant associations with self-esteem and optimism. With SOC added to Model 3, the significant association with physical activity limitations was unchanged, but the significant association with life stressors disappeared. Mental health was significantly associated with SOC.

Model 3 (full model) revealed the strongest association between mental health and SOC in both areas (urban A: β = −.30, p < .001; rural B: β = −.41, p < .001).

## Discussion

Regarding regional characteristics, social capital was significantly greater in rural B. According to the 2001 Cabinet Office Survey, social capital is likely to be less in big cities such as Tokyo and Osaka and higher in the countryside. The results of the current study reflect this reported trend. Participation in regional activities was significantly higher in rural B. Activities in that area typically had a close connection with the local region, such as neighborhood associations, neighborhood cleaning, and helping with ceremonial events. In urban A, activities were typically centered on individual roles and activities, such as clubs and parent–teacher associations. These findings show that social resources for rural B residents were closely dependent on the region where they lived. In contrast, urban A residents were more likely to obtain social resources more centered on individual roles and not closely related to the region. The same trends in a rural compared with an urban area have been reported by a previous study [18].

Urban A residents showed significantly higher self-esteem and optimism than rural B residents. This suggests that high self-esteem and optimism are a part of urban personality characteristics. Urban residents are likely to be more individualistic than rural residents. Rural residents tend to have more relatives in their vicinity and are likely to be more collectivistic than urban residents. Coping strategies were found to be different among urban and rural residents. Not only psychological resources, but also the structure of social resources, such as support networks and community activities, can be different as well [35]. The results of the current study reflect these reported characteristics.

As shown in Table 2, age, physical activity limitations, and economic status showed significant associations with SOC in both areas. Some studies have reported that education and SOC are directly associated [15,36]. However, another study indicated that higher education has an indirect effect on SOC through the occupation obtained after finishing schooling [36]. In the current study, economic status was found to be significantly associated with SOC in both areas. Although there was no direct association between education and SOC, education and economic status were associated with each other. It is reasonable to suppose that education affected economic status, and then SOC.

As for social resources, social capital and social support were significantly associated with SOC in both areas, with social capital more strongly associated in rural B than in urban A. It is probable that situation-related social capital holdings affected the degree of association with SOC. This seems to be reflected by regional characteristics of rural B. Participation in community activities was significantly associated with SOC in urban A, but not in rural B. Participation in community activities such as neighborhood associations and ceremonial events were an obligatory part of living in rural B. Almost 80% of the rural residents participated in such activities. It is possible, however, that these activities did not always have positive meaning for rural residents. In contrast, there were no community activities for urban A residents unless they were willing to participate in such activities. Therefore, participation in community activities in urban A might have had more positive meaning, and therefore was significantly associated with SOC.

As for psychological resources, self-esteem was most significantly associated with SOC in both areas. Optimism was significantly associated with SOC in urban A, but not in rural B. No significant association of humor was found in the urban or rural area. As for psychological resources, a previous study [16] indicated associations of SOC with optimism and sense of humor. In the current study, there was a strong association between self-esteem and SOC, and self-esteem was significantly associated with optimism and humor. When self-esteem was adjusted, there was no significant association of SOC with optimism and humor. It is probable that psychological resources had the strongest explanatory power related to SOC because self-esteem was most strongly associated. The explanatory power of demographic characteristics was stronger in urban A than in rural B, but that of social resources in rural B was stronger than in urban A, which confirms the results of a previous study [18]. The association of SOC and resources reflected situation-related resource holdings in each area.

SOC showed the most significant association with mental health in both areas. Mental health (Table 3) showed significant associations with physical activity limitations and life stressors in both areas, but the associations were weakened when social and psychological resources and SOC were added, which demonstrated their buffering effect on the negative influence of life stressors on health. When SOC was added, the association of self-esteem with mental health disappeared in both areas, but optimism in urban A and humor in rural B independently showed direct associations with mental health. SOC has been demonstrated to have a strong association with self-esteem [16,17] and is believed to be associated with mental health as an intermediary. The present results clearly show an association between better mental health and SOC. That is, the stronger SOC was, the less was the incidence of poorer mental health, consistent with findings in previous studies [11-13].

**Table 3:** Resources associated with mental health among urban and rural respondents

	**Urban A (n=363)**						**Rural B (n=269)**					
	**Model 1**		**Model 2**		**Model 3**		**Model 1**		**Model 2**		**Model 3**	
	β	p	β	p	β	p	β	p	β	p	β	p
Sociodemographic characteristics												
Gender (1=Male, 0=Female)	.06	.350	.07	.240	.03	.536	-.08	.097	-.07	.160	-.05	.291
Age (years)	-.24	<.001	-.20	<.001	-.13	.037	-.04	.461	-.03	.624	.02	.649
Physical activity limitations	.32	<.001	.29	<.001	.25	<.001	.33	<.001	.30	<.001	.28	<.001
Education												
Junior high school (ref)												
High school	.05	.688	.08	.522	.11	.362	-.01	.941	.00	.948	.08	.225
Junior or technical college	.00	.989	.08	.526	.11	.376	-.06	.415	-.02	.774	.05	.393
Four-year university or graduate school	.02	.912	.13	.341	.14	.270	.03	.609	.05	.389	.09	.107
Economic status	-.15	.015	-.06	.301	-.05	.402	-.06	.250	-.01	.891	-.01	.903
Marital status	.03	.573	.09	.095	.09	.094	-.06	.241	-.04	.382	-.06	.201
Employment status	-.10	.086	-.09	.082	-.08	.133	.00	.959	.01	.890	.02	.613
Life stressors	.27	<.001	.19	.003	.15	.015	.32	<.001	.22	<.001	.09	.085
Social resources												
Social capital			.05	.362	.04	.402			-.02	.640	.01	.761
Social support			.00	.958	.02	.672			-.07	.184	-.04	.391
Community activities			-.03	.626	.00	.940			-.02	.670	-.04	.358
Psychological resources												
Self-esteem			-.26	<.001	-.12	.088			-.21	<.001	-.05	.373
Humor			-.06	.289	-.05	.312			-.11	.020	-.10	.023
Optimism			-.16	.012	-.13	.029			-.08	.174	-.07	.163
SOC score					-.30	<.001					-.41	<.001
Adjusted R^2^	.36		.46		.49		.31		.40		.49	
ΔR^2^			.11		.04				.10		.09	

According to hierarchical multiple regression analysis, mental health (GHQ) score as dependent variable.

The current study shows that resources available to urban and rural residents are characteristic of the region where they live, and that social and psychological resources, as well as SOC, are associated with positive health. SOC was found to be significantly associated with social and psychological resources in both areas, playing an important function in mediating resources for better mental health. These findings confirm premises in Antonovsky’s salutogenic model and represent a valuable contribution of the current research.

In this study, response rates were relatively low, and the two groups of respondents were selected from only one urban and one rural region, which may limit the generalizability of our results. Further study is necessary, involving a more comprehensive population sample, with particular attention to assessing the reproducibility or significance of our findings. The focus of the current study was regional differences. However, it is anticipated and recommended that future studies make comparisons with previous studies by incorporating gender and age differences as well [16,19,37].

## Conclusions

The current study confirmed that resources available to urban and rural residents are characteristic of the area where they live, and that social and psychological resources, as well as SOC, are associated with better mental health. For the practical purpose of promoting good health of residents in a region, it is important to strengthen social resources in that region or compensate for resources that are lacking. Participation in regional activities may have more special, positive meaning for urban residents than for rural residents. It is possible that new social resources for urban residents can be cultivated by increasing their individual involvement with the region. Possible interventions might include facilitating exchange with neighbors among urban residents. Rural residents showed greater social capital and a higher rate of participation in community activities. In addition, those who had strong attachment to or confidence in their region were likely to have high SOC and better health. It is desirable that residents maintain such ties with their region. It will be important that not only participation in regional activities, but also the qualities of the resources be examined as to whether they are in fact the resources leading to better mental health. Possible interventions might include facilitating humor among urban residents and promoting optimism among rural residents. Health strategy that takes into account also the promotion of psychological resources is expected.

This study examined associations among health resources, SOC, and mental health in two socioculturally different areas. Possible directions for health support strategies, which reflect regional characteristics, were suggested.

## Competing interests

The authors declare that they have no competing interests.

## Authors’ contributions

Both authors contributed to the research design, interpretation of data, and drafts of the paper. YT performed the statistical analyses. All authors read and approved the final manuscript.

## Pre-publication history

The pre-publication history for this paper can be accessed here:

http://www.biomedcentral.com/1471-2458/12/1107/prepub
